# Osteoblasts are inherently programmed to repel sensory innervation

**DOI:** 10.1038/s41413-020-0096-1

**Published:** 2020-05-13

**Authors:** Luís Leitão, Estrela Neto, Francisco Conceição, Ana Monteiro, Marina Couto, Cecília J. Alves, Daniela M. Sousa, Meriem Lamghari

**Affiliations:** 1 Instituto de Investigação e Inovação em Saúde (i3S), Universidade do Porto, 4200-135 Porto, Portugal; 2 Instituto de Engenharia Biomédica (INEB), Universidade do Porto, 4200-135 Porto, Portugal; 3 Instituto de Ciências Biomédicas Abel Salazar (ICBAS), Universidade do Porto, 4050-313 Porto, Portugal

**Keywords:** Bone, Neurophysiology, Homeostasis

## Abstract

Tissue innervation is a complex process controlled by the expression profile of signaling molecules secreted by tissue-resident cells that dictate the growth and guidance of axons. Sensory innervation is part of the neuronal network of the bone tissue with a defined spatiotemporal occurrence during bone development. Yet, the current understanding of the mechanisms regulating the map of sensory innervation in the bone tissue is still limited. Here, we demonstrated that differentiation of human mesenchymal stem cells to osteoblasts leads to a marked impairment of their ability to promote axonal growth, evidenced under sensory neurons and osteoblastic-lineage cells crosstalk. The mechanisms by which osteoblast lineage cells provide this nonpermissive environment for axons include paracrine-induced repulsion and loss of neurotrophic factors expression. We identified a drastic reduction of NGF and BDNF production and stimulation of Sema3A, Wnt4, and Shh expression culminating at late stage of OB differentiation. We noted a correlation between Shh expression profile, OB differentiation stages, and OB-mediated axonal repulsion. Blockade of Shh activity and signaling reversed the repulsive action of osteoblasts on sensory axons. Finally, to strengthen our model, we localized the expression of Shh by osteoblasts in bone tissue. Overall, our findings provide evidence that the signaling profile associated with osteoblast phenotype differentiating program can regulate the patterning of sensory innervation, and highlight osteoblast-derived Shh as an essential player in this cue-induced regulation.

## Introduction

Peripheral innervation is a critical component of tissues’ structure and function. Neuronal signaling has been implicated as a regulatory mechanism of tissue homeostasis and regeneration.^1–6^ During the development of the peripheral nervous system, neurons project axons to reach their target tissues and form functional circuits. The amount, type, and patterning of innervation is achieved through the tissue-specific expression in space and time of attractive or repulsive axonal guidance molecules.^7,8^ Axonal terminals have the molecular mechanisms to accurately react to these guidance cues, ultimately ensuring the establishment of intricate patterns of neuronal networks.^7,8^

An increasing body of evidence has indicated the neuro-skeletal liaison as an important regulatory mechanism for bone development, turnover, and regeneration.^5,9^ In pathological scenarios such as fracture, bone cancer, or osteoporosis, where a deregulation of the bone homeostasis and/or regeneration processes occurs, changes in the pattern of bone innervation are also often observed, suggesting a disturbance in the neuronal signaling to the bone.^10–14^ In fact, the healthy bone is highly innervated by primary afferent sensory and sympathetic fibers, branching densely in the periosteum and, to a lesser extent, mineralized bone, and bone marrow.^9,15^ Importantly, anatomical mapping of innervation during skeletal development shows that sensory nerve fibers are the first to be detected in the bone microenvironment, particularly in areas with high osteogenic activity,^16–18^ which has attracted particular interest concerning bone formation.^19–23^ Indeed, the osteoprogenitor mesenchymal stem cells (MSC) have been reported to support neuronal survival and promote axonal outgrowth and regeneration through the expression of neurotrophic factors.^5,24–26^ In the development of mouse femur, nerve growth factor (NGF) expressed by MSC has been described as a skeletal neurotrophin, promoting and directing the outgrowth of sensory axons to primary and secondary centers of incipient ossification.^27^ However, the role of MSC-derived mature osteoblasts (OB) in controling sensory innervation in the bone is still unclear. OB have been described to promote axonal growth through the expression of NGF when submitted to mechanical loading, but not under static conditions,^28^ which might be an indication that OB no longer have the ability to control bone innervation. Therefore, it remains unknown whether the signaling profile associated with the OB phenotype differentiating program impacts the patterning of sensory innervation in bone.

In this study, we analyzed the paracrine signaling of OB-lineage cells throughout osteoblastogenesis and correlated it with sensory axonal behavior. We show that differentiation of human MSC to OB phenotype leads to marked impairment of their ability to promote axonal growth and creates a nonpermissive environment for the sensory nerve fibers through the expression of axonal repulsive cues. Overall, we provide valuable data on the contribution of OB-lineage cells in the regulation and maintenance of innervation in the bone.

## Results

### The secretome of OB-lineage cells impairs the development of sensory axonal networks

To explore if the MSC commitment to OB alters its neurotrophic ability, we exposed dorsal root ganglia (DRG) to the secretome of OB at different times of differentiation. OB were differentiated from MSC as previously described,^29^ and the conditioned medium was collected at day 0 (MSC CM), day 7 (D7 OB CM), day 14 (D14 OB CM), and day 21 (D21 OB CM). Under osteoinductive conditions, MSC showed transient expression of the early OB marker alkaline phosphatase and acquired the full OB phenotype after 21 days in culture, characterized by high osteocalcin gene expression levels and intense calcium deposition (Supplementary Fig. 1).

**Fig. 1:**
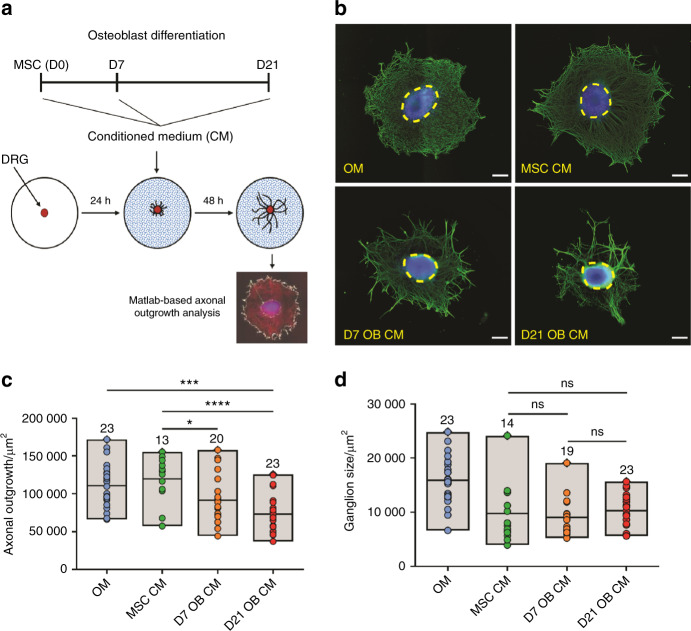
DRG exposed to the secretome of OB-lineage cells have reduced axonal networks. **a** Scheme of the experimental setup and timeline employed in these experiments. **b** Representative images of the axonal network of DRG after 48 h exposure to osteogenic medium (OM), and conditioned medium of undifferentiated (MSC CM), immature (D7 OB CM), and mature OB (D21 OB CM) (blue—DAPI; green—βIII-tubulin; dashed circles—DRG core; scale bar—200 µm). Quantification of the area of axonal outgrowth (**c**) and the core of the ganglion (**d**) in the different conditions. Results are presented as floating bar graphs (line represents the mean value, and the number above each bar represents the number of ganglia that were analyzed; *P < 0.05, ***P < 0.001, ****P < 0.000 1, ns non-significant)

The neurotrophic potential of the distinct conditioned media was tested on organotypic explants of DRG cultures, and the axonal outgrowth calculated using a Matlab-based algorithm^30^ (Fig. 1a). Our results showed that DRG treated with the conditioned medium of differentiating OB have significantly smaller axonal networks when compared with undifferentiated MSC. DRG treated with D7 OB and D21 OB CM exhibited a 17% and 34% reduction on the axonal mesh area, respectively, when compared with MSC CM, suggesting a gradual loss of the axonal growth potential with the maturation stage of OB (Fig. 1b, c). A significant decrease was also observed when comparing D21 OB CM with the nonconditioned OM (Fig. 1b, c). This is an important internal control, as the standard medium of a DRG culture is strikingly different from the culture medium used to differentiate OB. Hence, the observed effect was due to the conditioning of differentiated OB and not due to the culture medium itself.

In addition to the cell bodies of primary sensory neurons, DRG also encloses satellite cells and Schwann cells, that can provide support to axonal outgrowth.^31,32^ To discard a possible effect of the OB conditioned medium on the ability of nonneuronal cells to migrate from the ganglion core to periphery to support axonal outgrowth, we analyzed the area of the DRG core after treatment with the different secretomes (Fig. 1b, dashed yellow circles). No statistically significant differences in the area of the ganglion core were observed between DRG exposed to MSC, D7 OB, and D21 OB CM, indicating that cellular migration of nonneuronal cells from the DRG core was equivalent across the different secretomes of OB-lineage cells. These observations indicate that the reduction in the size of the axonal network with the CM of differentiating OB was not due to deficient supportability from DRG nonneuronal cells, and also that the different secretomes might trigger a localized effect on axons (Fig. 1d).

Altogether, these results suggest that the secretome of differentiating OB impair the development of the axonal network of sensory neurons, an effect more evident for the conditioned medium of OB at late stages of differentiation.

### The secretome of mature OB triggers repulsion of sensory axons

**Fig. 2:**
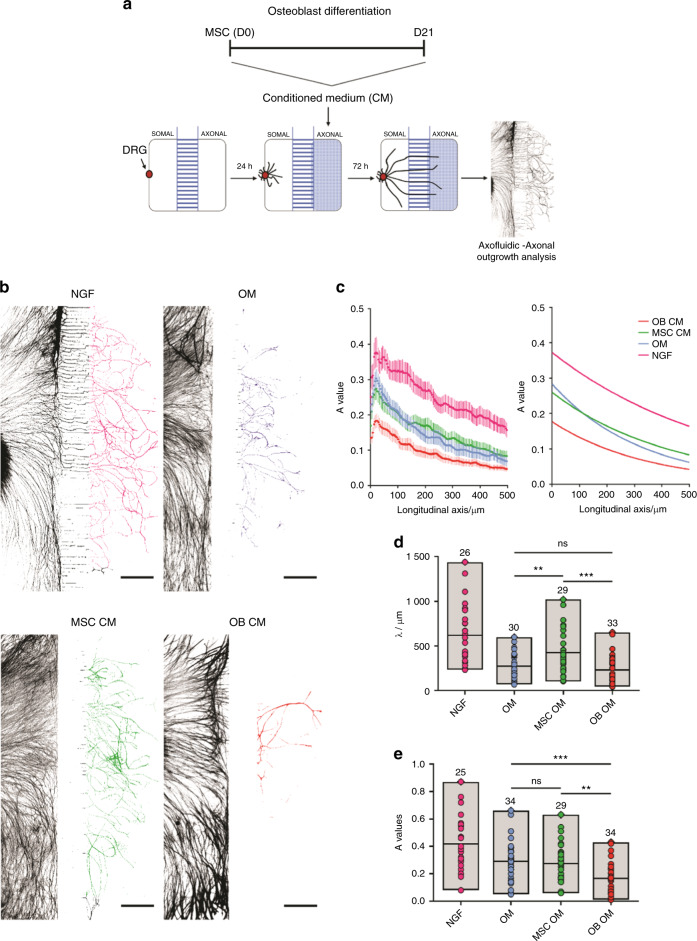
The secretome of mature OB applied locally to sensory axons, alters their behavior, and impairs their growth. **a** Scheme of the experimental setup and timeline employed in these experiments. **b** Representative images used for the quantification of the axonal growth upon 72 h exposure to standard neuronal growth medium containing NGF (positive control), osteogenic medium (OM), undifferentiated (MSC CM), and mature osteoblasts (OB CM) conditioned medium (axons stained against βIII-tubulin; scale bar—500 µm). **c** The combined plot of the data obtained using Axofluidic from all of the quantified microfluidic devices in the different conditions (left), and corresponding graphical representation of the exponential model (right), described by the spatial dependence decay function in the form $f\left( x \right)\,=\,A\,\times\,\exp ( - x/\lambda )$, that best fits the data. *A* value represents the amount of axons that cross the microgrooves and reach the axonal side, whereas in the longitudinal axis is represented the distance from the microgrooves (in µm). **d**, **e** Graphical representation of all *A* values (**c**) and *λ* values (**d**) in the different conditions. Results are presented as floating bar graphs (line represents the mean value, and the number above each bar represents the number of microfluidic devices that were analyzed; **P < 0.01; ***P < 0.001, ns non-significant)

To evaluate if the paracrine signaling of differentiating OB produces a localized effect in the axonal terminals of sensory neurons, we cultured DRG in microfluidic platforms that closely mimics an in vivo scenario, by allowing spatial and fluidic separation of the DRG core from distal axons^33,34^ (Fig. 2a). The reduced height of the microgrooves, combined with a higher volume on the somal compartment of the microfluidic devices, creates a slow yet sustained unidirectional flow of liquid from the somal to the axonal compartment. This ensures the retention of soluble components present in the OB CM in the axonal compartment, therefore producing a localized effect on axons.^33^

By employing this experimental design, we observed well-defined differences in the automatic curve fitting for spatial decay within the axonal compartment for the conditioned medium of OB differentiated for 21 days (OB CM), when compared with MSC CM, OM, and the standard neuronal growth medium containing NGF (Fig. 2c). Analysis of axonal outgrowth showed that axons stimulated with the secretome of OB were significantly shorter (lower *λ* values), and linger with values similar to the nonconditioned OM, in comparison with MSC CM (Fig. 2d). These results suggest that the MSC ability to promote axonal growth is lost with the progression of OB differentiation. Also, significantly fewer axons effectively crossed the microgrooves (lower *A* values) for the secretome of mature OB when compared with both MSC CM and OM. Moreover, no significant differences were found between MSC CM and OM conditions (Fig. 2e). These results suggest that soluble factors secreted by mature OB (and absent in the MSC CM) are not permissive for the axonal pathfinding and are possibly triggering a repulsive mechanism in the axons.

Taken together, our observations strongly indicate that as osteoblastogenesis progresses, MSC lose their ability to promote axonal growth, while triggering the establishment of a nonpermissive and repulsive environment for axonal growth.

### The OB secretome does not alter the expression of CGRP by sensory axons

**Fig. 3:**
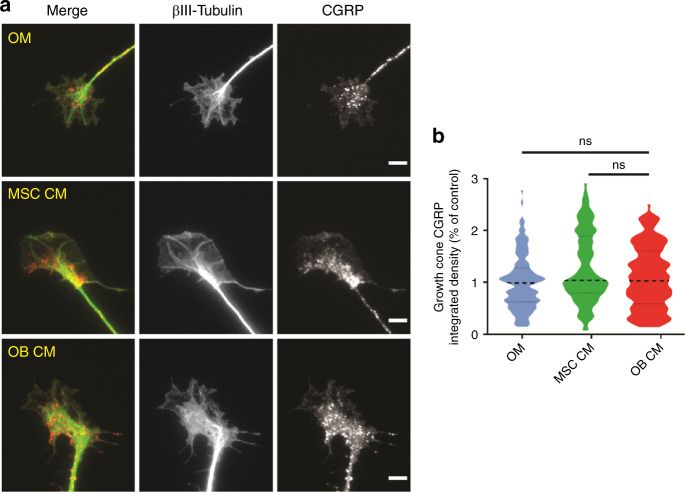
The secretome of OB-lineage cells does not impact the expression of CGRP at the growth cones of sensory neurons. **a** Representative images of the sensory growth cones exposed to osteogenic medium (OM), undifferentiated (MSC CM), and mature osteoblasts (OB CM) conditioned medium for 72 h (green—βIII-tubulin; red—CGRP; scale bar—5 µm). **b** Graphical representation of the integrated intensity of CGRP in different conditions. Results are presented as violin plot (middle dashed line represents the median value; upper and lower dashed lines represent the quartiles; ns non-significant)

The sensory–skeletal communication is achieved through the expression and release of the neurotransmitters calcitonin gene-related peptide (CGRP) and substance P by axonal terminals. Since our results strongly suggest that the secretome of mature OB produces a localized and repulsive effect on sensory axons, we evaluated if it also impairs CGRP expression. We employed the experimental design depicted in Fig. 2a, and measured the total fluorescence levels of CGRP at the growth cones, the highly motile and dynamic structures present at the distal end of axons (Fig. 3). Our analysis demonstrated no significant differences in the levels of CGRP in axonal terminals exposed to the secretome of mature OB (OB CM) and undifferentiated MSC (MSC CM) (Fig. 3a, b). These results suggest that the mechanisms underlying sensory neuropeptide expression are not affected by the secretome of differentiating OB.

### The OB-sensory neurons coculture replicates the secretome-induced axonal repulsion

**Fig. 4:**
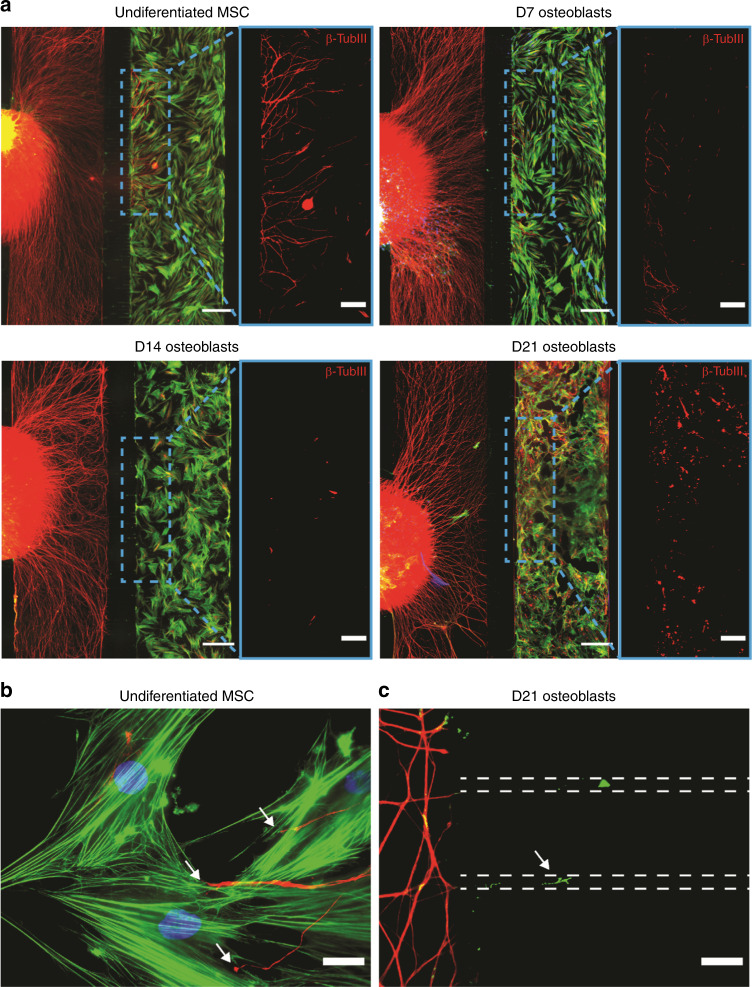
Mature OB prevent the growth of sensory axons in a coculture setup in compartmentalized microfluidic devices. **a** Coculture of DRG with OB-lineage cells at different time points of differentiation, i.e., undifferentiated MSC (top left), with 7 (top right), 14 (bottom left), and 21 (bottom right) days of differentiation (green—F-Actin; red—βIII-tubulin; blue—DAPI; scale bar—500 µm). Of notice, the progressive reduction in the number of axons in the OB-lineage cell compartment throughout OB differentiation. For better visualization of the axons present in the axonal compartment, a region was selected (dashed blue line) showing only the axons (red—βIII-tubulin; scale bar—200 µm). **b** Sensory axons growing and contacting undifferentiated MSC (white arrow, scale bar—25 µm). **c** Axons (white arrow) entering the microgrooves of the microfluidic device without reaching the OB compartment in the coculture setting of DRG and OB with 21 days of differentiation (dashed lines represent the microgrooves. Scale bar—25 µm)

To evaluate if the crosstalk between OB-lineage cells and sensory axons recapitulates the observed effect of OB on the axonal growth and repulsion, we performed a coculture of DRG with OB at different stages of differentiation, in compartmentalized microfluidic devices (Fig. 4). After 4 days in coculture, we observed axons growing interspersed into the compartment containing undifferentiated MSC (Fig. 4a—top left, b). Axons were also observed in the compartment containing OB differentiated for 7 days, however to a lesser extent when compared with MSC (Fig. 4a—top right). Importantly, axons were unable to grow towards both OB differentiated for 14 and 21 days (Fig. 4a—bottom left and right). We observed that axons entered the microgrooves of the microfluidic device but were incapable of crossing them, remaining close to the DRG compartment of the microfluidic (Fig. 4c). Taken together, these results are in line with our previous observations and strengthen our hypothesis that the commitment of MSC to OB creates a nonpermissive and repulsive environment for the development of an axonal network.

### The secretome of OB is depleted of neurotrophins and enriched in axonal repulsive molecules

To understand the molecular mechanisms underlying the axonal behavior mediated by OB-lineage cells, both in terms of elongation and repulsion, we analyzed the expression levels of neurotrophic factors and of proteins with a consolidated capability in promoting axonal repulsion at different stages of OB differentiation.

**Fig. 5:**
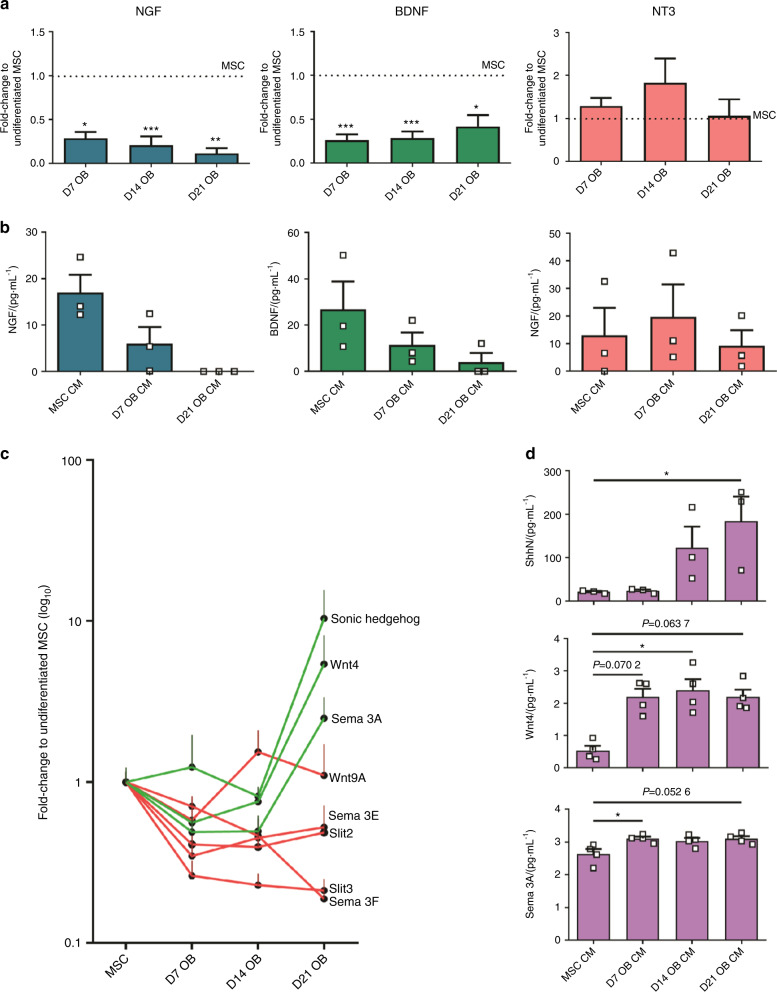
OB differentiation downregulates the expression of neurotrophic factors and upregulates the expression of axonal repulsive cues. **a** Gene expression analysis of NGF, BDNF, and NT-3. Values normalized to the values of undifferentiated MSC (horizontal dashed line). **b** ELISA analysis of NGF, BDNF, and NT-3 in the conditioned medium of OB at different stages of differentiation. **c** Fold-change variation in the gene expression of the selected axonal repulsive cues throughout OB differentiation. Values normalized to the values of undifferentiated MSC. Red lines depict the proteins that downregulated or showed no significant changes. Green lines depict the proteins that upregulated. **d** ELISA analysis of Shh, Sema3A, and Wnt4 in the conditioned medium of OB at different stages of differentiation. *P < 0.05; **P < 0.01; ***P < 0.001

Moreover, MSC have been described to promote axonal growth of DRG and to express a wide range of neurotrophic factors, including NGF, brain-derived neurotrophic factor (BDNF), and neurotrophin-3 (NT-3).^35–39^ In accordance, we observed the higher levels of both NGF and BDNF in the undifferentiated MSC, analyzed both by gene expression and the protein content in the conditioned medium (Fig. 5a, b). Interestingly, the expression levels of both neurotrophic factors showed a significant decrease throughout OB differentiation (Fig. 5a, b). This reduction was evident as soon as day 7 of OB differentiation, with the lowest levels of both NGF and BDNF observed for mature OB (after 21 days of differentiation). No significant changes were observed both in the gene expression and protein levels of NT-3 throughout OB differentiation.

We also quantified the expression of genes that encode secreted proteins that have been previously described to repel axons. The genes included were: Sonic Hedgehog (Shh), Wnt4, Wnt9A, Semaphorin 3A (Sema3A), Semaphorin 3E, Semaphorin 3F, Slit 1, Slit 2, and Slit 3. The expression profile of the analyzed repulsive cues changed throughout OB differentiation (Fig. 5c), indicating that Shh, Wnt4, and Sema3A are upregulated, and Slit 2, Slit 3, Semaphorin 3E, Semaphorin 3F, and Wnt9A are downregulated or showing no significant changes. Slit 1 was not detected at any time point (data not shown). Based on these results, we selected Shh, Wnt4, and Sema3A as possible players mediating axonal repulsion induced by the secretome of mature OB (Fig. 5d). In accordance, protein quantification showed that all selected repulsive cues were increased in the conditioned medium of differentiating OB. However, only Shh showed a gradual increase with the advance of OB differentiation, with higher expression levels by day 21 of differentiation, whereas both the Wnt4 and Sema3A profiles were characterized by a significant increase in the onset of OB differentiation, remaining stable afterward until day 21.

Overall these results confirm that MSC cease to produce neurotrophic factors throughout OB differentiation, namely NGF and BDNF, while upregulating the expression of axonal repulsive cues. The combined changes in the secretome of mature OB provides a hostile microenvironment for axonal growth, hindering the development of axonal network.

### OB-derived Shh contributes to sensory axonal repulsion

Shh is a well-established protein with the ability to promote axonal repulsion of projections from the central nervous system.^40,41^ Considering that our results show that axonal repulsion induced by OB-lineage cells increases with osteoblastic maturation (Figs. 1 and 4) and that Shh has a critical role in bone formation, healing, and aging (44–46), we have proceeded our study to assess the contribution of Shh in the OB-induced repulsion of sensory axons.

**Fig. 6:**
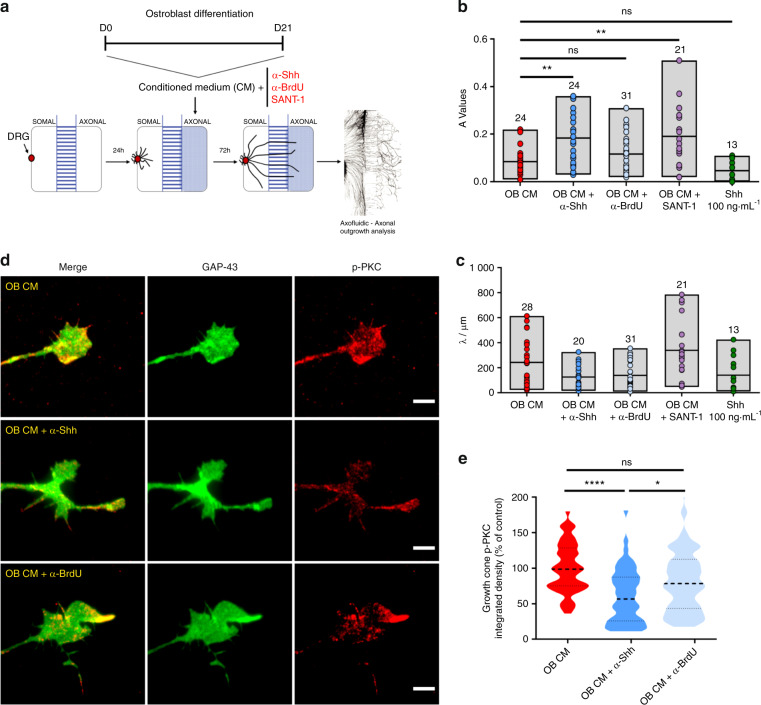
OB-derived Shh contributes to sensory axonal repulsion. **a** Scheme of the experimental setup and timeline employed in these experiments. **b**, **c** Graphical representation of *A* values (**a**) and *λ* values (**b**) for OB CM alone or supplemented with the Shh activity blocker antibody 5E1, the isotype control antibody anti-BrdU (G3G4), the Smo antagonist SANT-1, and the positive control Shh diluted in OM. Results are presented as floating bar graphs (line represents the mean value, and the number above each bar represents the number of microfluidic devices that were analyzed; **P < 0.01; ns non-significant). **d** Representative images of the sensory growth cones exposed for 10 min to mature osteoblasts (OB CM) alone, and supplemented with the 5E1 and G3G4 antibodies (green—GAP-43; red—phosphorylated PKCα; scale bar—5 µm). **e** Graphical representation of the integrated intensity of phosphorylated PKCα in different conditions. Results are presented as violin plot (middle dashed line represents the median value; upper and lower dashed lines represent the quartiles; ****P < 0.000 1, *P < 0.05, ns non-significant)

We supplemented the conditioned medium of mature OB (OB CM) with the monoclonal anti-Shh antibody 5E1 (Fig. 6a), known for its ability to block Shh binding to the receptor Patched1, thereby inhibiting Shh signaling.^42^ We observed that a significantly higher number of axons entered the axonal compartment of the microfluidic (higher *A* values) when Shh activity was blocked, as compared with both OB CM alone or the isotype control (Fig. 6b). No changes in the overall length of the axons were observed (Fig. 6c). With this approach, we were able to confirm that Shh is involved in the axonal repulsion induced by the OB CM.

Shh-induced axonal repulsion at the optic chiasm of contralateral retinal ganglion cells has been described to be dependent on the transmembrane Smoothened (Smo) protein, a critical activator of the Shh signaling pathway, and the downstream phosphorylation of protein kinase Cα (PKCα).^43,44^ To strengthen our hypothesis on the involvement of Shh signaling in the OB-induced axonal repulsion of sensory axons, we next asked whether the Smo protein is critical for this process and if phosphorylation of PKCα also occurs at the growth cones. The supplementation of the OB CM with the Smo antagonist SANT-1 also resulted in an increased amount of sensory axons at the axonal compartment (Fig. 6a–c), indicating a role for Smo in the sensory repulsion induced by the OB secretome. Shh has been shown to induce commissural axon growth cone turning within 10 min,^45^ and phosphorylation of PKCα after 2 min of stimuli.^44^ To maximize our experimental readout, we allowed sensory axons to accumulate in the axonal compartment, and specifically stimulated them for 10 min with OB CM alone, or supplemented with either the Shh-activity blocker 5E1 or its respective isotype control (anti-BrdU). We observed significantly higher PKCα phosphorylation levels in the OB CM and isotype control setting when compared with the condition where Shh activity was blocked (Fig. 6d, e).

Together, our results demonstrate that Shh expressed by OB contributes to sensory axon repulsion, in a mechanism that is Smo-dependent and requires the phosphorylation of PKCα at the growth cone.

### Shh co-localizes with OB-lineage cells in the developing bone

Cumulative evidences demonstrated that sensory innervation is detected during embryonic bone development, in close association with osteogenic events.^27^ In addition, Shh has been described to play a role in OB differentiation,^46–49^ but its expression in bone microenvironment by OB-lineage cells remains to be elucidated. Therefore, to evaluate in vivo if Shh is expressed in developing bone, we performed immunohistochemical analysis in the E16.5 mice femurs.

**Fig. 7:**
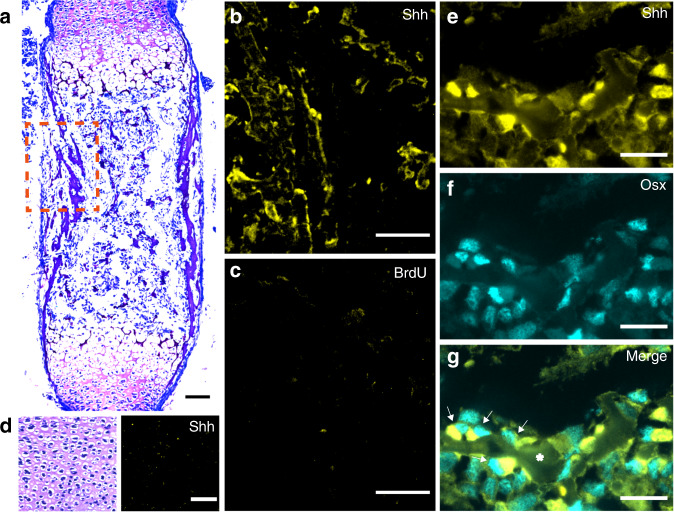
Shh co-localizes with OB-lineage cells in primary ossification centers. **a** Histochemical slice of an embryonic mice femur, stained with toluidine blue (scale bar—50 µm). **b**, **c** Amplification of the region highlighted in **a** immunostained for anti-Shh (**b**) and negative control anti-BrdU (**c**); scale bar—50 µm. **d** Region of hyaline cartilage showing no expression of Shh (scale bar—50 µm). **e**–**g** Amplification of a trabecular region from **a**, depicting co-localization of Shh labeling with osterix-positive OB-lineage cells (white arrows); scale bar—20 µm

Longitudinal bone cross-sections of E16.5 femurs revealed that Shh was highly expressed adjacent to sites of incipient mineralization in the primary ossification center, co-localizing with trabecular bone surfaces (Fig. 7a–c). Of note, no Shh expression was observed in the hyaline cartilage at the growth plate (Fig. 7d). Furthermore, an immunohistochemistry co-staining was performed against Osterix/Sp7 (Osx) and Shh to identify OB-lineage cells expressing Shh. Indeed, the immunohistochemistry analysis demonstrated a high degree of co-localization between Osx-positive OB-lineage cells and Shh in trabecular bone surfaces at the ossification center of developing femurs (Fig. 7e–g). Overall, these results show that Shh is actively expressed in vivo in the bone microenvironment from the early stages of bone development and sensory innervation, and co-localizes with OB-lineage cells at sites of new bone formation.

## Discussion

**Fig. 8:**
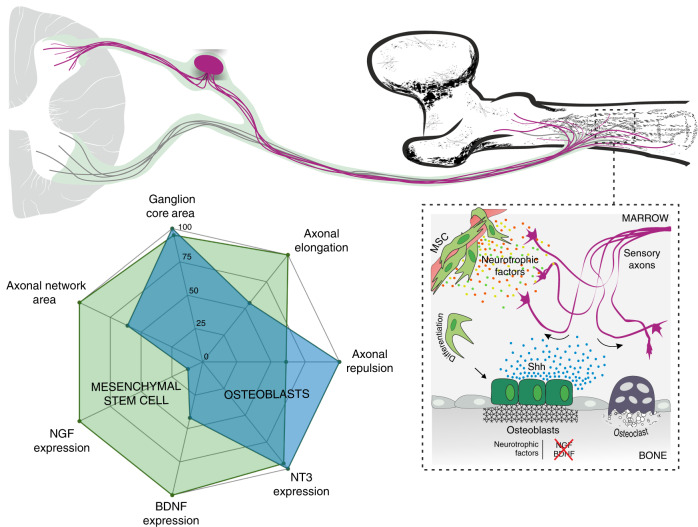
Overall model summarizing the remarkable ability mature OB have to repel and impair the growth of sensory axons

In this study, we demonstrated that the differentiation of human MSC to OB phenotype leads to marked impairment of their ability to promote axonal growth. This was evidenced under sensory neurons and OB-lineage crosstalk. The mechanisms by which OB-lineage cells provide this nonpermissive environment for axons include paracrine-induced repulsion and loss of neurotrophic factors expression. Among the signaling molecules secreted by OB-lineage cells, we identified a drastic reduction of NGF and BDNF production and stimulation of Sema3A, Wnt4, and Shh expression that culminates at late stage of OB differentiation. We noted a synchronized relationship between Shh expression profile, OB differentiation stages, and OB-mediated axonal repulsion. By blocking Shh activity and signaling via immunodepletion and pharmacological antagonism, respectively, we were able to reverse the repulsive action of OB on sensory axons. To consolidate our results, we localized the expression of Shh by OB in bone tissue (Fig. 8).

We and others have reported significant alterations in the density and distribution of nerve fibers in musculoskeletal pathophysiology.^10–12,50^ Bone disorders such as Paget’s disease, osteoporosis, fibrous dysplasia, and osteogenesis imperfecta are frequently associated with pain, suggesting a disturbance in the sensory signaling.^10^ Also, mice models of non-healed fractures show exuberant sensory sprouting at lesion sites,^11^ and in bone cancer, an abnormal outgrowth of sensory projections is observed in the marrow, mineralized bone, and periosteum.^12,14,51^ These pathological outcomes are associated with a deregulation of the normal activity of cells from the bone microenvironment and consequent aberrant expression of pro-inflammatory cytokines and factors that promote axonal growth.^26,50^

The maintenance of the pattern of innervation in the bone throughout adulthood and the role of mature and functional OB in this process are mechanisms still poorly understood. In homeostatic conditions, the distribution and density of sensory axons in the bone tissue are defined by the combinatory action of both attractive and repulsive molecules, ultimately shaping the network of sensory innervation. Our study shows that, under homeostatic settings, OB create a nonpermissive and repulsive environment for sensory axonal growth, without compromising the expression of the sensory neuropeptide CGRP at the growth cone. In accordance, anatomical mapping of nerve fibers in the adult bone revealed no physical contact between sensory nerve fibers and OB.^52^

Our analysis of neurotrophic factors demonstrated that MSC commitment to OB triggers an almost immediate interruption of the expression of NGF and BDNF, and a progressive loss of neurotrophin levels in the secretome throughout OB differentiation. Both NGF and BDNF are important mediators of axonal elongation, and active members in the bone microenvironment.^39,53^ In the developing mouse femur, sensory axons are directed to bone-forming sites in a precise spatiotemporal manner, in a symbiotic cross-communication with osteoprogenitor cells.^27^ NGF produced by osteoprogenitor cells directs sensory axons to sites of incipient ossification, and the consequent activation of sensory nerve terminals results in the release of osteogenic cues.^27^ Interestingly, the loss-of-function we observed for osteoprogenitors regarding the expression of neurotrophic factors does not seem to be irreversible. NGF expression has been demonstrated in OB from mice ulnas within 3 h after mechanical loading, decreasing afterward and absent in non-loaded limbs, suggesting that OB can reactivate the expression of NGF in response to mechanical loads to promote further sensory innervation and consequently stimulate bone formation.^28^ This information strengthens our findings that mature OB do not express NGF and do not promote axonal outgrowth under homeostatic conditions. They also highlight the intrinsic ability of OB to regulate bone innervation and patterning, by switching on and off the expression of sensory nerve attractants to bone-forming sites.

In our study, we cultured sensory neurons in microfluidic platforms that resembles the anatomical in vivo scenery. This experimental setup allowed us to witness that fewer axons sprouted towards the OB conditioned microenvironment, suggesting that paracrine signals from OB-origin trigger a repulsion mechanism in sensory axons. Indeed, our analysis of known axonal repulsive cues confirmed this hypothesis and showed that their expression profile changed throughout OB differentiation. Sema3A and Wnt4 were upregulated at the onset of MSC commitment to OB phenotype, whereas Shh expression increased throughout osteoblastogenesis and was concomitant with our observations of axonal repulsion under the paracrine effect of OB-lineage cells. Discrepancies observed between gene and protein levels have been reported previously, and might be due to posttranscriptional mechanisms or lack of availability of the translation machinery.^54^ Shh is an important player that controls growth and patterning of different embryonic structures including the spinal cord and skeleton.^55,56^ Importantly, it has been described to have a critical role in bone in promoting osteogenic differentiation.^46–49^ OB are responsive to Shh signaling at bone fracture sites undergoing intense remodeling, which has been shown to regulate proliferation and differentiation.^47^ Also, it has been described that Shh is specifically expressed in CGRP^+^ sensory fibers innervating both the epithelial and mesenchymal compartments of the mouse incisor, and that its activity promotes the proliferation and enamel and dentin formation.^57^ Here, we propose that Shh secreted by OB is a major repulsive player of sensory axons, by inducing axonal repulsion in a Smo- and PKCα- dependent signaling mechanism. Indeed, blockage of both Shh activity and Smo receptor signaling in vitro resulted in the ablation of axonal repulsion induced by OB. We also demonstrate in vivo that Shh is highly expressed adjacent to sites of incipient mineralization in the primary ossification center, co-localizing with Osx-positive OB-lineage cells at trabecular bone surfaces. These observations are in line with previous reports that showed that systemic overexpression of Shh in mice increases OB number and trabecular number in the bone marrow cavity of the femoral diaphysis.^58^ In addition, sensory projections have been observed extending into the limb and terminating near the perichondrial region of the femur as early as embryonic day 14.5.^16,27^ Importantly, we detected Shh at embryonic day 16.5, indicating that Shh expression coincides with the presence of sensory nerve fibers in the bone microenvironment, and suggesting that Shh might regulate skeletal innervation in vivo from the early stages of bone development. Nevertheless, in the context of this study, and since Sema3A and Wnt4 were also elevated in the secretome of OB-lineage cells, we cannot exclude the possibility of combined action these proteins in the axonal repulsion induced by OB. Shh has been shown to indirectly affect axonal guidance of commissural neurons by establishing a gradient of the Wnt antagonist Sfrp1 (Secreted frizzled-related protein 1), and consequently regulate Wnt function.^59^ Also, Shh activates the repulsive response of commissural axons to semaphorins, allowing for the correct exit of commissural axons from the floorplate.^41^

Both OB and osteoclasts are required for sustaining bone homeostasis, by maintaining a balance between resorption and formation. In addition, a broad range of literature has attributed to the sensory signaling a role in promoting bone formation.^20–23^ Importantly, a direct link between osteoclast activity and sensory axonal outgrowth has been recently described.^26^ Zhu et al. reported in osteoarthritic mice that aberrant osteoclast activity over-induce sensory axonal outgrowth on the subchondral bone through the secretion of the axonal attractant Netrin-1.^26^ Our results in vitro and under homeostatic conditions show that once OB mature to their full phenotype, they divert sensory projections from sites of incipient bone formation. We hypothesize that, under homeostatic conditions, osteoclasts might also control sensory innervation by attracting nerve fibers to sites with higher osteoclast activity and consequent bone degradation, therefore indirectly promoting bone formation. Overall, we suggest a new model in which the combined activity of osteoclasts and OB not only sustain bone homeostasis but also control sensory patterning in the bone, resulting in a simultaneous, and possibly correlated, balance of bone mass and innervation.

In conclusion, our results unravel new mechanisms underlying the communication between OB and sensory terminals and strengthen the importance of OB in the control of bone innervation. Moreover, we also shed light on new key players in the neuro-bone communication, how their expression changes throughout OB differentiation, and how they can impact innervation. With this new information, studies aiming to manipulate OB activity, and consequently change its secretome, can be employed to gain further insights on the bone-peripheral nervous system communication. An accurate picture of this crosstalk is of the utmost importance, and will ultimately allow researchers and clinicians to unravel new candidates as potential targets, not only for bone pain therapies, but also to control the neuropathological outcomes resulting from the disturbances in bone cell activity.

## Materials and methods

### Human MSC culture and OB differentiation

MSC (Lonza) were expanded in Dulbecco’s modified Eagle (DMEM) medium with low glucose (Gibco), supplemented with 10% v/v fetal bovine serum (FBS) qualified for MSC expansion (Gibco), and 1% v/v penicillin/streptomycin (P/S) (Gibco). Cells were used between passages 6 and 10. For OB differentiation, MSC were plated in 24-well plates at a density of 25 000 cells/well. Cultures were left undisturbed for 3 days at 37 °C in a 5% CO_2_ humidified incubator to allow MSC to reach confluency. Osteogenesis was induced by replacing the expansion medium with osteogenic medium (OM), composed of DMEM with low glucose (Gibco), supplemented with 10% v/v FBS, 1% v/v P/S, 100 nmol·L^−1^ Dexamethasone (Sigma-Aldrich), 10 μmol·L^−1^ β-glycerophosphate (Sigma), and 50 μmol·L^−1^ 2-phospho-L-ascorbic acid (Sigma-Aldrich). The OM was replaced every 2–3 days for 21 days. To collect the conditioned medium, OB-lineage cells were incubated in 300 µL of OM and left undisturbed for 3 days at 37 °C in a 5% CO_2_ humidified incubator. Afterward the medium was filtrated and stored at −80 °C until further use.

### Cultures of embryonic DRG

Primary cultures of mice DRG were prepared from E16.5 C57BL/6 embryos, as previously described.^60^ All animal procedures were approved by the i3S ethics committee and by the Portuguese Agency for Animal Welfare (Direção-Geral de Alimentação e Veterinária), in compliance with the European Community Council Directive of September 22, 2010 (2010/63/UE). Briefly, pregnant females were sacrificed by cervical dislocation, and the embryos immediately decapitated. Embryonic ganglia were reached through the dorsal side of the embryo after spinal cord removal under the stereoscopic magnifier. DRG were retrieved from the lumbar regions L1–L6. The meninges were cleaned from the isolated DRG, and the roots were cut. DRG were randomly distributed for all experimental conditions, and cultured with neurobasal medium supplemented with 2% v/v B-27 Serum-Free Supplement® (Invitrogen), 60 mol·L^−1^ 5-fluoro-2′-deoxyuridine (Sigma-Aldrich), 25 mmol·L^−1^ glucose (Sigma-Aldrich), 1 mmol·L^−1^ pyruvate (Sigma-Aldrich), 50 ng·mL^−1^ 7 S NGF (Calbiochem), 2 mmol·L^−1^ glutamine (BioWhittaker), and 1% P/S.

### DRG exposure to OB-lineage cell secretome

#### (a) Global exposure in organotypic cultures

DRG were seeded into the lower wells of a 15-well μ-Slide Angiogenesis plate (IBIDI) embedded in a fibrin hydrogel, as previously described.^60^ DRG cultures were left undisturbed for 24 h at 37 °C in a 5% CO_2_ humidified incubator. At this time, the medium was completely replaced with the conditioned medium from the different experimental conditions, including nonconditioned OM. Cultures were then left undisturbed for an additional 48 h at 37 °C in a 5% CO_2_ humidified incubator.

#### (b) Axonal-specific exposure in microfluidic devices

For the experiments using microfluidic devices, DRG were plated in glass slides coated with 0.1 mg·mL^−1^ poly-D-Lysine (PDL) (Sigma-Aldrich) overnight at 37 °C and laminin 5 μg·mL^−1^ (Sigma-Aldrich) as previously described.^34^ Commercially available microfluidic devices (Merck Millipore and Xona Microfluidics) were adapted for explant DRG culture and assembled as previously described.^61^ Cultures were left undisturbed for 24 h. At this time, the medium from the axonal side was substituted by the conditioned medium of the different experimental conditions, including nonconditioned OM, and the culture was left undisturbed for an additional 72 h. A volume difference between the ganglia and axonal compartment (≈150 µL) was maintained throughout this period to prevent the diffusion of the conditioned medium from the axonal to the somal side (Fig. 2a). The higher volume on the somal side causes a slow net flow of liquid from the somal to the axonal compartment, thus ensuring that the conditioned medium was restricted to the axonal compartment.^33^

### Coculture in microfluidic platforms

MSC were plated in microfluidic devices assembled in glass slides coated with 0.1 mg·mL^−1^ PDL overnight at 37 °C and laminin 5 μg·mL^−1^ as previously described.^34^ MSC were plated at a density of 3 000 cells/device and allowed to expand for 3 days. Afterward, osteogenesis was induced, and cells were allowed to differentiate for 7, 14, and 21 days. DRG were plated as described above, and the coculture was left undisturbed for 4 days at 37 °C in a 5% CO_2_ humidified incubator. A volume difference between the OB-lineage cells compartment and DRG compartment (≈150 µL) was maintained throughout this period to allow the diffusion of the OB conditioned medium towards the DRG side.

### Blockage of Shh activity

Functional activity of Shh secreted by OB was blocked by adding the 5E1 antibody (DSHB (deposited to the DSHB by Jessell, T.M./Brenner-Morton, S.t)) or the nonspecific control antibody G3G4 (DSHB (deposited to the DSHB by Kaufman, S.J.)) to the conditioned medium at a final concentration of 6 μg·mL^−1^. Before stimuli, 5E1 and G3G4 were purified using Protein G HP Spintrap (GE Healthcare) according to the manufacturer’s protocol. For the experiments aiming to assess the contribution of the Shh receptor Smo in the axonal repulsion-mediated by OB, the SMO receptor antagonist SANT-1 (Merck Millipore) was added to the conditioned medium at a final concentration of 250 ng·mL^−1^. Exogenous Human Sonic Hedgehog (Sigma) was used as a positive control for mice sensory axon repulsion, and added to the conditioned medium at a final concentration of 100 ng·mL^−1^. In all cases, the culture was left undisturbed for 72 h at 37 °C in a 5% CO_2_ humidified incubator.

To assess the phosphorylation status of PKCα in growth cones, the DRG culture was initially left undisturbed for 4 days, for axons to accumulate in the axonal compartment. At this time point, a starving period was performed only in the axonal compartment with plain Neurobasal medium for 5 h. Throughout the starving period, a volume difference between the axonal compartment and the somal compartment (≈150 μL) was maintained to prevent the diffusion of the complete medium from the somal to the axonal side. The conditioned medium of mature OB was concentrated ten times by centrifugation at 12 000 rpm, at 4 °C, and for 75 min, using 3 kDa MW cutoff filter units (Sartorius Stedim biotech). Axons were stimulated for 10 min with the concentrated conditioned medium diluted 1:10 in plain Neurobasal medium, with and without the 5E1 and G3G4 antibodies at a final concentration of 6 μg·mL^−1^, and immediately fixed afterward.

### Immunocytochemistry, image acquisition, and quantification

DRG cultures were fixed with 2% PFA/2% sucrose for 10 min, followed by an additional 30 min with 4% of PFA/4% sucrose in phosphate-buffered saline (PBS) at room temperature (RT). Ganglia were simultaneously permeabilized and blocked for 30 min at RT in a solution consisting of 0.25% (v/v) Triton X-100 (Sigma-Aldrich), 5% v/v goat serum (GS) (Invitrogen), and 5% v/v FBS in PBS.

For the 3D organotypic cultures and the experiments to assess axonal outgrowth in microfluidic devices, DRG were incubated with a primary antibody directed against the neuronal-specific marker βIII-tubulin (Promega) diluted 1:2 000 in blocking solution (5% v/v GS and 5% v/v FBS in PBS), overnight at 4 °C. Afterward, cells were washed and incubated for 1 h at RT with the secondary antibody (Alexa Fluor 488, Invitrogen) and DAPI (Sigma-Aldrich), diluted 1:1 000 and 1:10 000, respectively, in blocking solution. Images were captured with IN Cell Analyzer 2000 equipped with IN Cell Investigator software (GE Healthcare) at the i3S BioSciences Screening Unit. Radial outgrowth, defined as the area between the ganglion edge and the outgrowth front, was determined. The outgrowth area was computed according to Bessa et al.^30^ To quantify axonal outgrowth in microfluidic platforms, neurite outgrowth was measured with AxoFluidic, an algorithm designed to quantify neurite projection within these platforms.^34^ The exponential fit was given by the spatial dependence decay function $f\left( x \right)\,=\,A\,\times\,\exp ( - x/\lambda )$, where the constant *A* represents the degree of axons that can effectively cross the microgrooves and enter the axonal compartment, and *λ* the scale of spatial decay, as a measure to represent the length of the neurites.^34^

For the analysis of CGRP at the growth cones, DRG were incubated with primary antibodies directed against the βIII-tubulin and CGRP (Sigma-Aldrich) diluted 1:2 000 and 1:6 000, respectively, in blocking solution, overnight at 4 °C. For the analysis of PKCα phosphorylation at the growth cones, DRG were incubated with primary antibodies directed against the GAP-43 (Abcam), and p-PKCα (Santa Cruz Biotechnology) diluted 1:1 000 and 1:250, respectively, in blocking solution, overnight at 4 °C. In both cases, afterward, cells were washed and incubated for 1 h at RT with the secondary antibodies (Alexa Fluor 488 and 568, Invitrogen) diluted 1:1 000, in blocking solution. Images were captured with a widefield inverted microscope DMI6000 FFW (Leica Microsystems) equipped with LAS X software (Leica Microsystems) at the i3S Advanced Light Microscopy Unit. Growth cones were randomly chosen, based on βIII-tubulin and GAP-43 fluorescence, without observation of CGRP and p-PKCα intensity, respectively. Total CGRP and p-PKCα fluorescence was measured with Image J software, and the background intensity of each image was subtracted. For each selected growth cone, we determined the total of CGRP and p-PKCα fluorescence per area. The values obtained per growth cone were normalized against the control mean of that single experiment.

### qRT-PCR analysis

Total RNA was extracted using the Direct-zol™ RNA miniPrep according to the manufacturer’s protocol (Zymo Research). RNA final concentration and purity (OD260/280) was determined using a NanoDrop 2000 instrument (NanoDrop Technologies). RNA was reverse transcribed into cDNA using the NZY First-Strand cDNA Synthesis Kit (NZYTech), according to the manufacturer’s protocol. For the analysis of repulsive cues, a personalized PrimePCR array (Bio-Rad Laboratories) was performed. qRT-PCR experiments were run using an iCycler iQ5 PCR thermal cycler (Bio-Rad Laboratories) and analyzed with the iCycler IQTM software (Bio-Rad). Target gene expression was quantified using the cycle threshold (Ct) values and relative mRNA expression levels were calculated as follows: 2^(Ct reference gene − Ct target gene). Human β-2-microglobulin was used as a reference gene. Both target and reference genes were amplified with efficiencies between 100% ± 5%.

### Immunohistochemistry

Femurs from E16.5 C57BL/6 embryos were harvested and fixed in 4% PFA at 4 °C for 24 h. After three washes in PBS, samples were processed for paraffin embedding. Three-micrometer-thick longitudinal cross-sections were obtained using a microtome (RM2255, Leica Biosystems) and stained for toluidine blue. For immunohistochemistry, tissue sections were deparaffinized and rehydrated before heat-induced antigen retrieval (98 °C, 10 mmol·L^−1^ citrate buffer, pH 6.0). Sections were simultaneously permeabilized and blocked for 1 h at RT in a solution consisting of 0.20% (v/v) Triton X-100, 10% v/v FBS, and 1% v/v BSA in PBS, and incubated with the primary antibody 5E1 (1:100), G3G4 (1:100), and anti-Sp7/Osterix (Abcam, 1:1 000) diluted in blocking solution overnight at 4 °C. Afterward sections were washed and incubated for 1 h at RT with the secondary antibodies (Alexa Fluor 488 and 568, Invitrogen) diluted 1:1 000, in blocking solution. Images were captured on an Axiovert 200 inverted microscope equipped with AxioVision 4.8 software (Zeiss) at the i3S Bioimaging Unit.

### ELISA analysis

The amount of NGF, BDNF, NT-3, and neurotrophin-4/5 in the conditioned medium of OB-lineage cells was quantified using a multi-neurotrophin rapid screening ELISA kit (Biosensis). For the repulsive cues, ELISA kits were used to detect and quantify the amount of Draxin (Raybiotech), Sonic Hedgehog (Raybiotech), Semaphorin 3A (ElabScience), and Wnt4 (Raybiotech). All procedures were performed according to the manufacturers’ protocol.

### Statistical analyses

All experiments were repeated at least three times. In all experiments, variables were subjected to the homogeneity of variance test to assess if they followed a normal distribution. Depending on whether they followed a normal distribution or not, a parametric two-way ANOVA or a nonparametric Kruskal–Wallis analysis was performed, respectively, to assess statistically significant differences. Differences between groups were considered statistically significant when *P < 0.05, **P < 0.01, ***P < 0.001. Data analysis was performed using GraphPad Prism (v8.02) for Windows.

## Supplementary information

Supplementary Information
