# Supplementary material for: Osteoblasts are inherently programmed to repel sensory innervation
Source: Bone Res. 2020 May 13;8:20. doi: 10.1038/s41413-020-0096-1 (PMC7220946; doi:10.1038/s41413-020-0096-1)
Supplement: Supplementary file 1 — Supplementary Information [file 41413_2020_96_MOESM1_ESM.docx]

**Supplementary Figure 1**


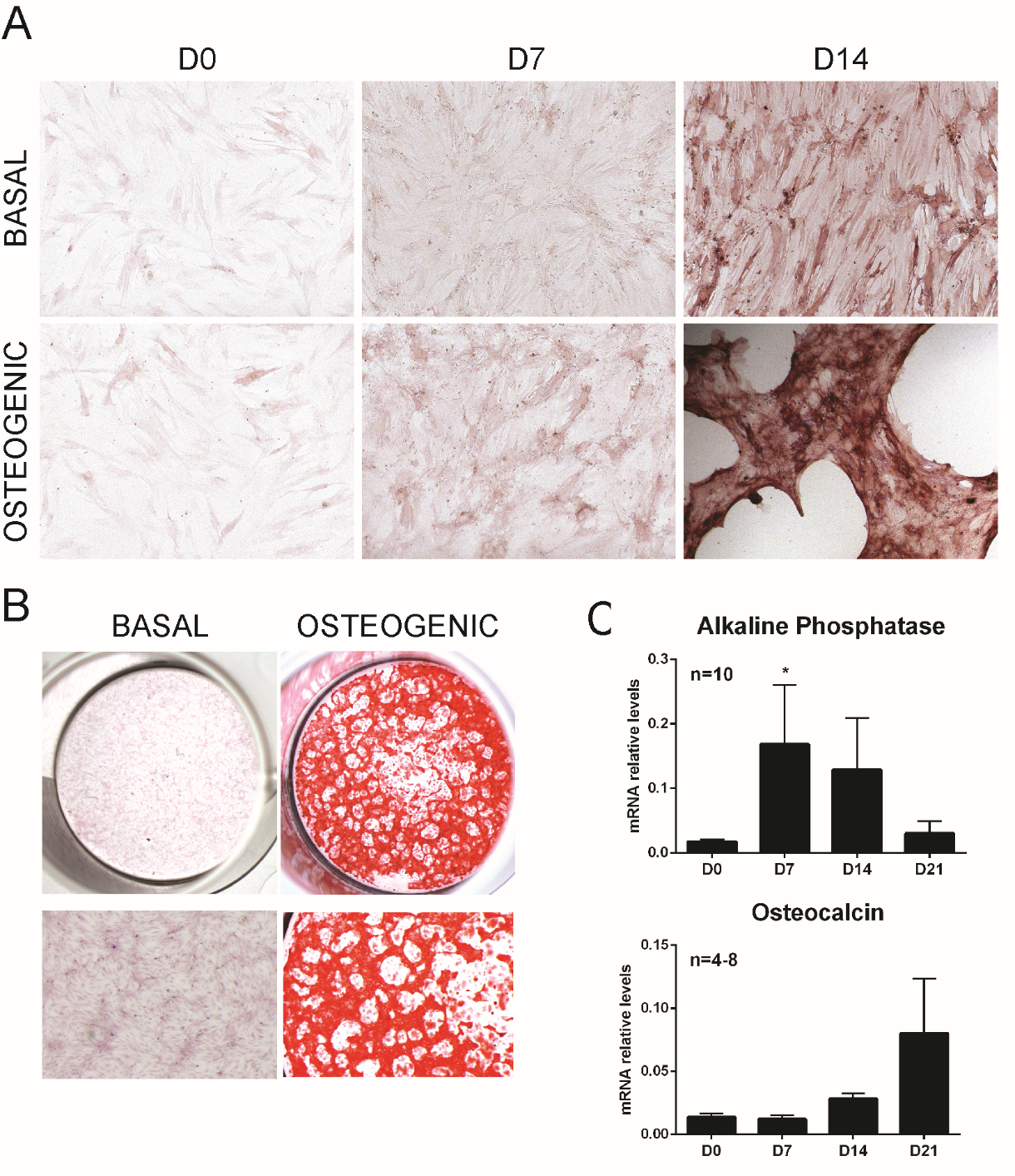


**Supplementary Figure 1 –** A) Representative images of the ALP cytochemical staining was performed in MSC at 7 and 14 days of culture. B) Representative images of Alizarin Red staining performed in MSC at 21 days of culture. C) Gene expression analysis of ALP and osteocalcin throughout 21 days of OB differentiation. (**P*<0.05).

**Supplementary Figure 2**

**
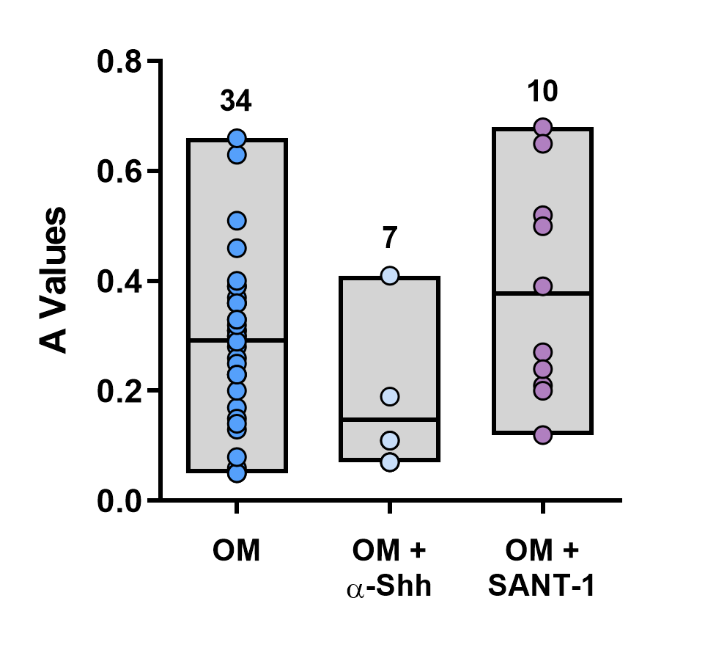
**

**Supplementary Figure 2 –** Graphical representation of *A* values for OM alone or supplemented with the Shh activity blocker antibody 5E1 and the Smo antagonist SANT-1. Results are presented as floating bar graphs (line represents the mean value, and the number above each bar represents the number of microfluidic devices that were analyzed;
